# Customized birth weight for gestational age standards: Perinatal mortality patterns are consistent with separate standards for males and females but not for blacks and whites

**DOI:** 10.1186/1471-2393-5-3

**Published:** 2005-02-20

**Authors:** K S Joseph, Russell Wilkins, Linda Dodds, Victoria M Allen, Arne Ohlsson, Sylvie Marcoux, Robert Liston

**Affiliations:** 1 Perinatal Epidemiology Research Unit, Departments of Obstetrics and Gynaecology and of Pediatrics, Dalhousie University, Halifax, Nova Scotia, Canada; 2 Health Analysis and Measurement Group, Statistics Canada, Ottawa, Ontario, Canada; 3 Division of Maternal-Fetal Medicine, Department of Obstetrics and Gynaecology, Dalhousie University, Halifax, Nova Scotia, Canada; 4 Departments of Paediatrics and of Obstetrics and Gynecology, University of Toronto, Toronto, Ontario, Canada; 5 Department of Social and Preventive Medicine, Université Laval, Sainte-Foy, Quebec, Canada; 6 Department of Obstetrics and Gynecology, University of British Columbia, Vancouver, British Columbia, Canada

## Abstract

**Background:**

Some currently available birth weight for gestational age standards are customized but others are not. We carried out a study to provide empirical justification for customizing such standards by sex and for whites and blacks in the United States.

**Methods:**

We studied all male and female singleton live births and stillbirths (22 or more weeks of gestation; 500 g birth weight or over) in the United States in 1997 and 1998. White and black singleton live births and stillbirths were also examined. Qualitative congruence between gestational age-specific growth restriction and perinatal mortality rates was used as the criterion for identifying the preferred standard.

**Results:**

The fetuses at risk approach showed that males had higher perinatal mortality rates at all gestational ages compared with females. Gestational age-specific growth restriction rates based on a sex-specific standard were qualitatively consistent with gestational age-specific perinatal mortality rates among males and females. However, growth restriction patterns among males and females based on a unisex standard could not be reconciled with perinatal mortality patterns. Use of a single standard for whites and blacks resulted in gestational age-specific growth restriction rates that were qualitatively congruent with patterns of perinatal mortality, while use of separate race-specific standards led to growth restriction patterns that were incompatible with patterns of perinatal mortality.

**Conclusion:**

Qualitative congruence between growth restriction and perinatal mortality patterns provides an outcome-based justification for sex-specific birth weight for gestational age standards but not for the available race-specific standards for blacks and whites in the United States.

## Background

Birth weight-specific perinatal mortality curves among male and female births intersect to produce a paradox: overall perinatal mortality rates and perinatal mortality rates at lower birth weights are relatively higher among male births, while at higher birth weights perinatal mortality rates are relatively higher among female births [1]. This puzzling observation reflects a general phenomenon that is also seen when birth weight- and gestational age-specific perinatal mortality curves are contrasted across race, plurality, maternal smoking status, parity, altitude, country, and other determinants of birth weight and gestational age [2-14]. We have previously presented a solution for this paradox of intersecting mortality curves that involves a reformulation of perinatal and neonatal mortality risk [15-20]. This reformulation, based on the fetuses at risk approach, eliminates the crossover phenomenon and provides several new insights into perinatal health issues.

In this paper, we demonstrate the paradoxical crossover of birth weight-specific perinatal mortality curves among male and female births and show how this phenomenon is resolved using the fetuses at risk approach. We also explore issues related to fetal growth restriction among males and females using the same approach. This latter issue is particularly important from a conceptual and clinical standpoint because the current literature on birth weight for gestational age standards (sometimes referred to as fetal growth standards) is confusing. Some standards provide unisex reference values [21-24], several are sex-specific [1,25-34] and yet others provide both sex-specific and unisex reference values [35-38]. Of equal concern is the fact that several standards are customized for different races [1,25,27-29], parity [25,27,29,34,36], plurality [24,30] and other characteristics [27], while others are not [21-23,26,31-33,35,37].

We used the fetuses at risk approach to contrast growth restriction and perinatal mortality rates among males and females in order to provide empirical justification for sex-specific (vs unisex) birth weight for gestational age standards. We also constructed and compared gestational age-specific growth restriction and perinatal mortality curves among whites vs blacks in order to evaluate currently available birth weight for gestational age standards (single standard vs separate standards for whites and blacks in the United States).

## Methods

We used data on all reported live births and stillbirths in the United States in 1997 and 1998 (National Center for Health Statistics perinatal mortality data file for all states and the District of Columbia for 1997 and 1998). Live births and infant death records for these years have been previously linked and gestational duration has been calculated based on the last menstrual period (LMP). Missing or inconsistent information on gestational age has been imputed or replaced in a small fraction (approximately 7 percent) of records by the National Center for Health Statistics (Hyattsville, Maryland). Gestational age was imputed from the month and year of the LMP when the exact LMP day was missing [39]. LMP-based gestational age information was replaced by the clinical estimate [40] when the former was inconsistent with birth weight or when there was no information on LMP (approximately 5 percent of births).

Analyses were restricted to singleton live births and stillbirths ≥22 weeks gestational age and ≥500 g birth weight in order to eliminate potential problems arising from regional differences in birth registration. Male and females births were first contrasted in terms of their gestational age and birth weight distributions. Birth weights were categorized into 500 g intervals for this purpose (500–999 g, 1,000–1,499 g, 1,500–1,999 g and so on). Birth weight-specific perinatal mortality rates, calculated within these birth weight categories, were computed as per convention by dividing the number of stillbirths and early neonatal (0 to 6 days) deaths in any birth weight category by the number of total births (stillbirths and live births) in that birth weight category. Similarly, gestational age-specific perinatal mortality rates among male and female births were contrasted, with rates computed by dividing perinatal deaths at any given gestation by the number of total births at that gestation.

The numbers of fetuses at risk for stillbirth and early neonatal death at each gestation were then used to calculate a second set of perinatal mortality rates. Under this fetuses at risk formulation, the stillbirth rate at 28 weeks gestation was computed by dividing the number of stillbirths at 28 weeks by the number of live births and stillbirths at 28 or more completed weeks of gestation. This implies that fetuses who delivered at 29, 30, 31 and 32 or more weeks gestation were also at risk of stillbirth at 28 weeks [15-19,41-44]. The fetuses at risk formulation applies equally to early neonatal death since a fetus (unborn) at 28 weeks gestation is at risk of birth and early neonatal death at that gestation [15,17,18]. Thus gestational age-specific perinatal/neonatal mortality rates under this formulation were calculated with perinatal/neonatal deaths at any gestational age in the numerator and the fetuses at risk of perinatal/neonatal death at that gestation in the denominator. This represents a survival analysis model with censoring of subjects (fetuses) at death or birth which ever occurs earlier (for a schematic depiction of the survival analysis model, see reference 18). In this model, neonatal death (and, in other contexts, serious pregnancy-related morbidity such as cerebral palsy [16]) is assigned to the point of birth since the responsible pathologic event/process is present at birth [18]. Gestational age-specific 'birth rates' (i.e., the number of births at any particular gestational week divided by the number of fetuses at risk of birth at that gestation) and rates of gestational age-specific labor induction/cesarean delivery were also estimated using the fetuses at risk approach [15-18].

We also examined gestational age-specific patterns of fetal growth restriction using the fetuses at risk approach [15,17-19]. The number of small-for-gestational age (SGA) live births at each gestation was divided by the number of fetuses at risk at that gestation in order to obtain the gestational age-specific SGA rate (or the gestational age-specific fetal growth restriction rate). SGA live births were identified using the 10^th ^percentile cut-off from a birth weight for gestational age standard based on live births in the United States [38]. Gestational age-specific SGA rates were calculated using both the unisex and sex-specific 10^th ^percentile values provided by this standard [38] to evaluate how well patterns of gestational age-specific growth restriction correspond with patterns of gestational age-specific perinatal mortality. This evaluation was premised on the belief that fetal growth restriction patterns should be qualitatively congruent with gestational age-specific perinatal mortality patterns. Such an expectation is consistent with clinical understanding and studies which show that growth restricted fetuses have a substantially higher perinatal mortality than appropriate-for-gestational age fetuses. For instance, Williams et al [1] showed that perinatal mortality at each gestational week was much higher among growth restricted births at the 10^th ^percentile of birth weight for gestational age (eg., perinatal mortality rate 138 per 1,000 total births at 34–35 weeks) compared with appropriate-for-gestational age births at the 50th percentile of birth weight for gestational age (eg., perinatal mortality rate 27 per 1,000 total births at 34–35 weeks). We also examined gestational age-specific growth restriction differences among males and females using rate ratios (eg., growth restriction rate among males at 35 weeks gestation divided by growth restriction rate among females at 35 weeks gestation) and contrasted these with gestational age-specific differences in stillbirth and neonatal mortality rates (also using rate ratios eg., stillbirth rate among males at 35 weeks divided by the stillbirth rate among females at 35 weeks; early neonatal death rate among males at 35 weeks divided by the early neonatal death rate among females at 35 weeks). This was done to ascertain the relationship between patterns of growth restriction and patterns in the two components of perinatal mortality (stillbirth and early neonatal death).

Comparisons of male and female gestational age-specific growth restriction and gestational age-specific perinatal mortality patterns were contrasted with similar comparisons according to maternal race. Specifically, live births and stillbirths ≥22 weeks of gestational age and ≥500 g birth weight in the United States in 1997 and 1998 were used to compare gestational age-specific growth restriction and perinatal mortality rates among whites vs blacks.

Identification of SGA live births among blacks and whites was carried out using a single standard for both races [38] and also a race-specific standard [29]. As with contrasts between males and females, the contrasts between whites and blacks were restricted to singleton births.

Differences in rates were assessed using rate ratios and excess risks. Taylor series 95% confidence intervals were calculated on all rate ratios. All p values presented are two-sided. Sensitivity analyses were carried out to assess the potential effect of gestational age errors on patterns of growth restriction and perinatal mortality among males and females. Specifically, we reassessed growth restriction and mortality patterns among males and females after excluding all births for whom menstrual-based gestational age was either imputed or replaced by the clinical estimate of gestation.

## Results

There were 3,905,694 singleton male births in the United States in 1997 and 1998 (≥22 weeks gestational age and ≥500 g birth weight). The low birth weight (<2,500 g) rate among male live births was 5.5%, and 10.5% of male live births were born preterm (<37 weeks). There were 3,723,153 female births in the United States during the same period and relative to males, female live births had a higher rate of low birth weight (6.4%, p < 0.0001) but a lower rate of preterm birth (9.4%, p < 0.0001). Males had a 14% (95% confidence interval 12 to 16, p < 0.0001) higher perinatal mortality than females; perinatal mortality rates among males and females were 6.78 and 5.95 per 1,000 total births, respectively.

**Figure 1:**
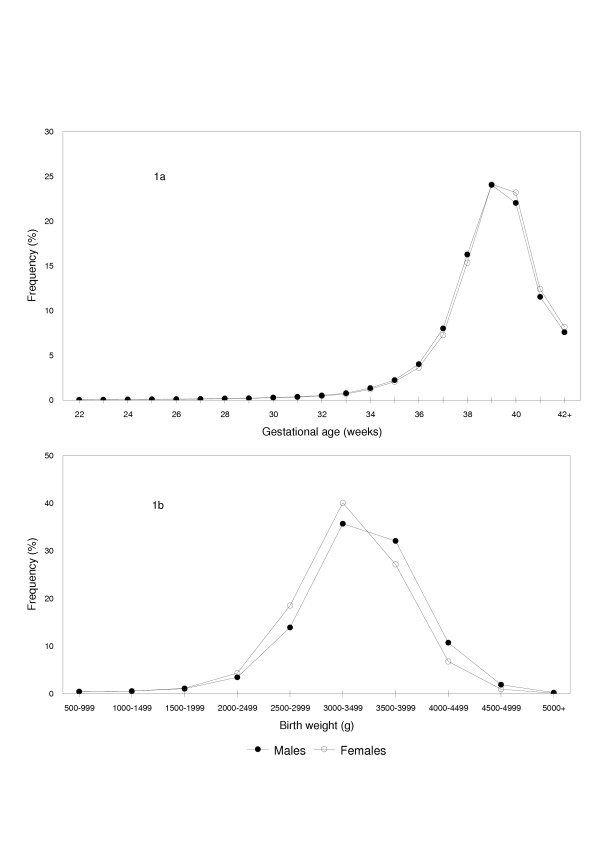
Gestational Age and Birth Weight Distributions of Male and Female Singleton Live Births. Gestational age (1a) and birth weight (1b) distributions of male and female singleton live births ≥22 weeks and ≥500 g in the United States, 1997 and 1998.

**Figure 2:**
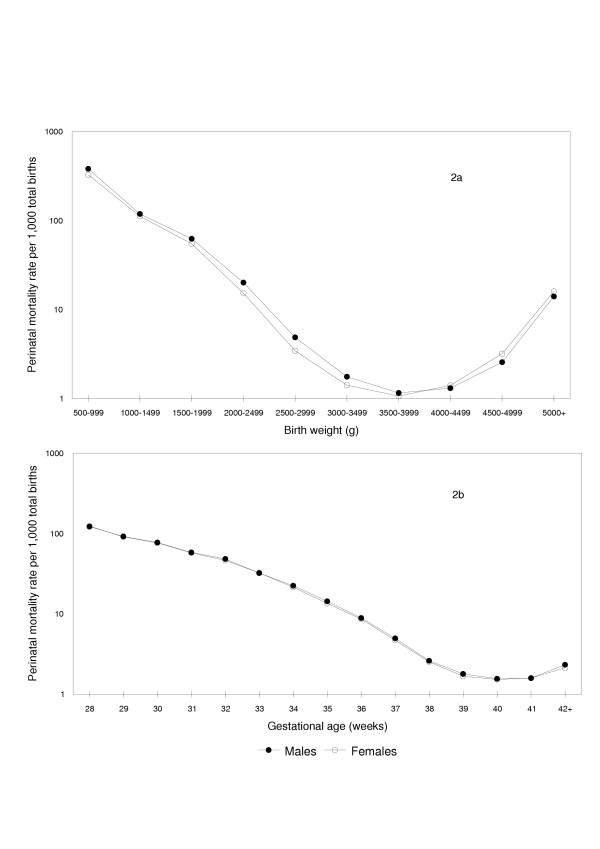
Conventional Calculation: Birth Weight- and Gestational Age-Specific Perinatal Mortality Rates among Male and Female Births. Conventional calculation: birth weight-specific (2a) and gestational age-specific (2b) perinatal mortality rates per 1,000 total births among male and female singleton births in the United States, 1997 and 1998.

The gestational age distribution of male live births (Figure 1) was 'shifted to the left' relative to female live births (p < 0.0001), while the birth weight distribution of females was markedly 'shifted to the left' relative to that of male live births (p < 0.0001). Birth weight-specific perinatal mortality rates (conventional calculation, perinatal deaths per 1,000 total births in a given birth weight category) showed the crossover paradox with males having relatively higher rates of perinatal death at birth weights <4,000 g, while females had relatively higher perinatal mortality rates at higher birth weights (Figure 2a). In contrast, gestational age-specific perinatal mortality rates (conventional calculation, perinatal deaths per 1,000 total births at any gestational week) showed similar mortality patterns among males and females (Tables 1 and 2), with males having a slightly higher perinatal mortality rate at some gestational ages (Figure 2b).

**Table 1:** Gestational Age-Specific Numbers and Rates of Perinatal Death among Male Singleton Births, United States, 1997 and 1998.

Gestational age	Stillbirths	Live births	Early neonatal deaths	Perinatal mortality rate (1)†	Fetuses at risk	Perinatal mortality rate (2)†
28	648	6,808	263	122.2	3,841,944	0.24
29	579	8,100	221	92.2	3,834,488	0.21
30	701	11,297	230	77.6	3,825,809	0.24
31	668	14,339	208	58.4	3,813,811	0.23
32	809	20,242	209	48.4	3,798,804	0.27
33	786	30,140	212	32.3	3,777,753	0.26
34	881	51,673	298	22.4	3,746,827	0.31
35	915	86,166	338	14.4	3,694,273	0.34
36	1,033	154,986	354	8.9	3,607,192	0.38
37	1,144	308,629	394	5.0	3,451,173	0.45
38	1,173	626,450	470	2.6	3,141,400	0.52
39	1,122	925,764	541	1.8	2,513,777	0.66
40	897	848,527	431	1.6	1,586,891	0.84
41	469	444,468	237	1.6	737,467	0.96
≥42*	454	292,076	229	2.3	292,530	2.33

Total‡	17,680	3,888,014	8,800	6.8	3,905,694	6.78

† Total births at each gestational week served as the denominator for perinatal mortality rates (1), while perinatal mortality rates (2) were calculated using fetuses at risk as the denominator (see text). All rates are expressed per 1,000.

**Figure 3:**
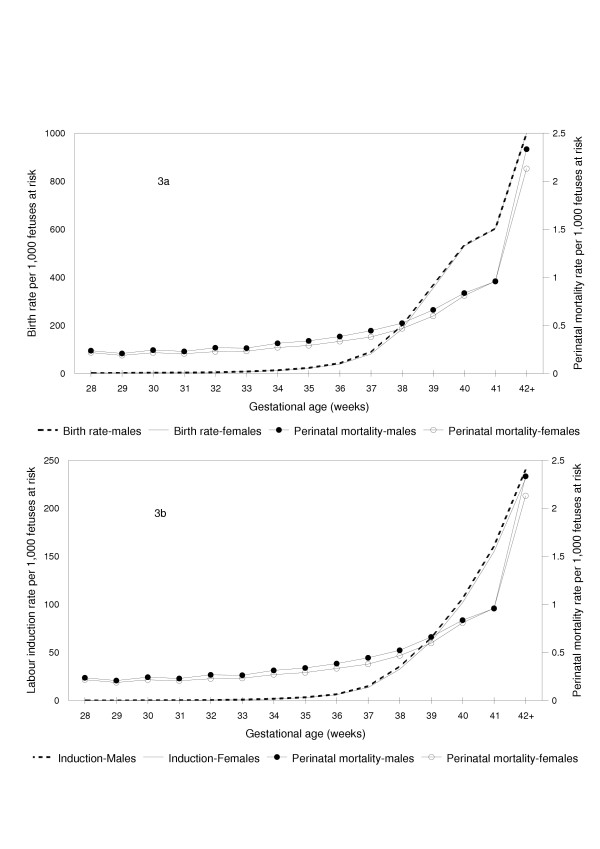
Fetuses at Risk Approach: Gestational Age-Specific Birth, Labor Induction and Perinatal Mortality Rates among Male and Female Births. Fetuses at risk approach: Gestational age-specific birth rates (3a, primary Y-axis), labor induction rates (3b, primary Y-axis) and perinatal mortality rates (3a and 3b, secondary Y-axis) among male and female singleton births in the United States, 1997 and 1998.

**Figure 4:**
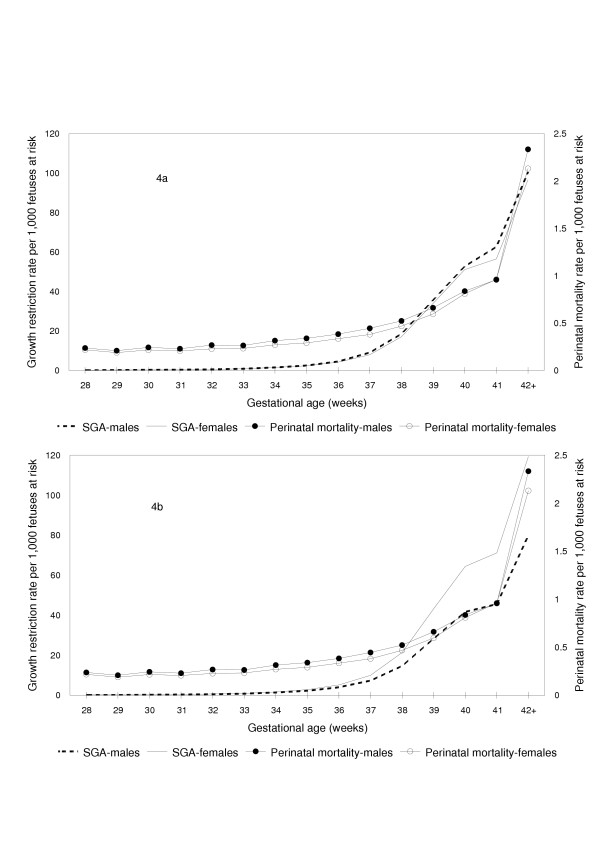
Fetuses at Risk Approach: Gestational Age-Specific Growth Restriction and Perinatal Mortality Rates among Male and Female Births. Fetuses at risk approach: Gestational age-specific fetal growth restriction (primary Y-axis) and perinatal mortality rates (secondary Y-axis) among male and female singleton births, with growth restriction rates based on sex-specific (4a) and unisex (4b) birth weight for gestational age standards, United States, 1997 and 1998.

**Figure 5:**
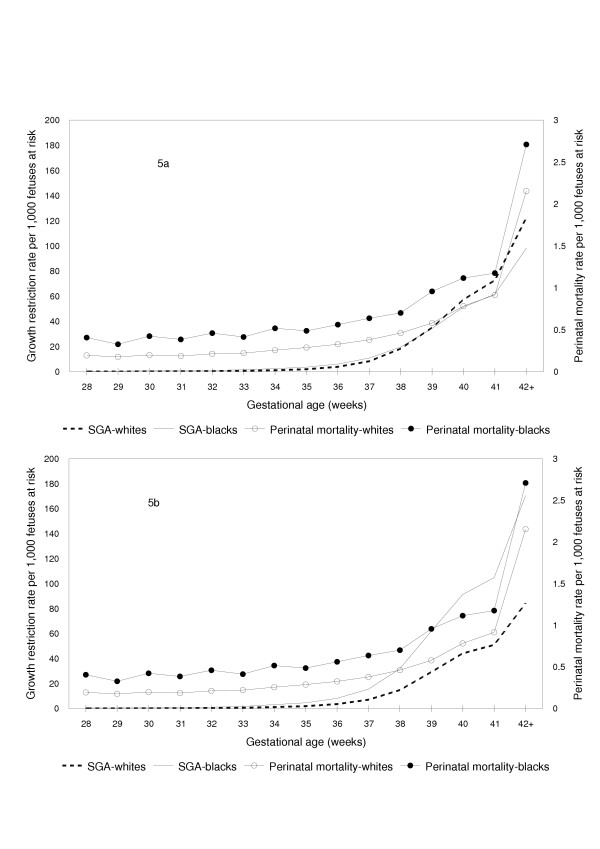
Fetuses at Risk Approach: Gestational Age-Specific Fetal Growth Restriction and Perinatal Mortality Rates among White and Black Births. Fetuses at risk approach: Gestational age-specific fetal growth restriction (primary Y-axis) and perinatal mortality rates (secondary Y-axis) among white and black singleton births, with growth restriction rates based on a race-specific standard (5a) and on a single birth weight for gestational age standard (5b), United States, 1997 and 1998.

* Large increase in perinatal mortality (2) at ≥42 weeks is partly because the period of risk exceeds 1 week (see also Figures 3-5).

‡ All gestational ages, including those ≥22 weeks and those with missing gestational age.

**Table 2:** Gestational Age-Specific Numbers and Rates of Perinatal Death among Female Singleton Births, United States, 1997 and 1998.

Gestational age	Stillbirths	Live births	Early neonatal deaths	Perinatal mortality rate (1)†	Fetuses at risk	Perinatal mortality rate (2)†
28	614	5,838	184	123.7	3,665,497	0.22
29	530	7,000	158	91.4	3,659,045	0.19
30	611	9,742	179	76.3	3,651,515	0.22
31	578	12,493	173	57.5	3,641,162	0.21
32	632	17,168	195	46.5	3,628,091	0.23
33	654	25,282	187	32.4	3,610,291	0.23
34	747	44,275	221	21.5	3,584,355	0.27
35	796	75,238	234	13.5	3,539,333	0.29
36	874	133,386	286	8.6	3,463,299	0.33
37	927	267,501	337	4.7	3,329,039	0.38
38	1,071	563,676	361	2.5	3,060,611	0.47
39	1,072	885,523	419	1.7	2,495,864	0.60
40	926	851,848	376	1.5	1,609,269	0.81
41	505	455,313	222	1.6	756,495	0.96
≥42*	443	300,234	198	2.1	300,677	2.13

Total‡	15,537	3,707,616	6,614	6.0	3,723,153	5.95

† Total births at each gestational week served as the denominator for perinatal mortality rates (1), while perinatal mortality rates (2) were calculated using fetuses at risk as the denominator (see text). All rates are expressed per 1,000.

* Large increase in perinatal mortality (2) at ≥42 weeks is partly because the period of risk exceeds 1 week (see also Figures 3-5).

‡All gestational ages, including those ≥22 weeks and those with missing gestational age.

Gestational age-specific perinatal mortality rates calculated using the fetuses at risk approach showed that perinatal mortality rates increased with increasing gestational age (Figure 3). Males had a higher perinatal mortality than females at virtually all gestational ages (Tables 1 and 2). Gestational age-specific 'birth rates' (Figure 3a), gestational age-specific labor induction rates (Figure 3b) and gestational age-specific labour induction and/or cesarean delivery rates (data not shown) were marginally (but consistently) higher among pregnancies with males as compared with pregnancies with females (Figure 3). For example, the birth rate among males at 35 weeks gestation was 23.6 per 1,000 fetuses at risk, while that among females at 35 weeks was 21.5 per 1,000 fetuses at risk (rate ratio 1.10, 95% confidence interval 1.09 to 1.11, p < 0.0001). The labour induction rates at 35 weeks among males and females were 3.6/1,000 and 3.1/1,000 fetuses at risk, respectively; rate ratio 1.10, 95% confidence interval 1.07 to 1.13, p < 0.0001.

Figure 4 compares gestational age-specific rates of fetal growth restriction among males and females. When growth restriction was determined using a sex-specific standard, growth restriction rates among males were higher than growth restriction rates among females at all gestational ages and this pattern was qualitatively congruent with sex differences in perinatal mortality (Figure 4a). For instance, males at 35 weeks gestation had an 8 percent (95% confidence interval 5 to 11, p < 0.0001) *higher *growth restriction rate than females at the same gestational week (sex-specific standard) and this was qualitatively congruent with a 17 percent (95% confidence interval 7 to 27 percent, p = 0.0003) *higher *perinatal death rate among males compared with females at 35 weeks gestation. On the other hand, when a unisex standard was used to identify growth restricted live births, males had a *lower *rate of growth restriction at all gestational ages and this was not qualitatively congruent with the *higher *gestational age-specific pattern of perinatal mortality among males (Figure 4b). For instance, at 35 weeks gestation, growth restriction rates determined using a single standard for both males and females showed that males had a 20 percent (95% confidence interval 18 to 22 percent, p < 0.0001) *lower *rate of growth restriction compared with females (not consistent with the 17% *higher *perinatal mortality rate).

Overall growth restriction rates based on a sex-specific standard showed that rates were 3% (95% CI 2 to 3) higher among males. Stillbirth and early neonatal mortality differences (rate ratios) among male vs female births both favored females (Table 3), although the mortality differences were much larger for early neonatal mortality (27%, 95% CI 23 to 31) than for stillbirth (8%, 95% CI 6 to 11). Gestational age-specific differences in growth restriction between males and females based on a sex-specific standard (eg., rate ratio at 35 weeks 1.08, 95% CI 1.05 to 1.11, Table 3) tended to be similar to gestational age-specific differences in stillbirth rates (eg., rate ratio at 35 weeks 1.10, 95% CI 1.00 to 1.21, Table 3), while differences in gestational age-specific early neonatal mortality tended to be larger (eg., rate ratio at 35 weeks 1.38, 95% CI 1.17 to 1.63, Table 3). Sensitivity analyses carried out to examine the potential effect of gestational age errors (by excluding births among whom gestational age was imputed or for whom the clinical estimate of gestation was used) showed essentially the same patterns of growth restriction and perinatal mortality among males and females.

**Table 3:** Gestational Age-Specific Rates of Fetal Growth Restriction Based on a Sex-Specific Standard [38] and Differences in Growth Restriction, Stillbirth and Early Neonatal Mortality Among Males and Females, Singleton Births, United States, 1997 and 1998.

Gestational age	Fetal growth restriction					Stillbirth rate ratio(males vs females)	Early neonatal mortality rate ratio(males vs females)
			
	Males		Females		Rate ratio (males vs females)		
					
	Number	Rate †	Number	Rate †			
28	631	0.2	502	0.1	1.20	1.01	1.36
29	764	0.2	660	0.2	1.10	1.04	1.33
30	1,165	0.3	923	0.3	1.20	1.10	1.23
31	1,539	0.4	1,369	0.4	1.07	1.10	1.15
32	2,141	0.6	1,866	0.5	1.09	1.22	1.02
33	3,294	0.9	2,946	0.9	1.07	1.15	1.08
34	5,691	1.6	5,098	1.5	1.07	1.13	1.29
35	8,934	2.5	7,922	2.3	1.08	1.10	1.38
36	15,813	4.6	13,910	4.2	1.09	1.13	1.19
37	30,029	9.1	25,180	7.9	1.15	1.19	1.13
38	55,401	18.5	49,599	17.1	1.09	1.07	1.27
39	84,257	35.7	79,440	33.9	1.05	1.04	1.28
40	75,983	52.8	74,504	51.1	1.03	0.98	1.16
41	36,956	62.6	34,214	56.5	1.11	0.95	1.10
≥42	14,679	100.5	14,551	96.7	1.04	1.05	1.19

Total‡	337,277	91.6	312,684	89.3	1.03	1.08	1.27

† Gestational age-specific growth restriction rates (based on a sex-specific standard [38]) were calculated by dividing the number of small-for-gestational age live births (<10^th ^percentile) at any gestational age by the number of fetuses at risk at that gestation. Stillbirth and early neonatal mortality rates were also calculated using fetuses at risk as the denominator.

‡ All gestational ages ≥22 weeks, except for growth restriction indices which were based on live births between 28 and 42 weeks.

Patterns of gestational age-specific growth restriction among whites and blacks could not be reconciled with patterns of gestational age-specific perinatal mortality, when growth restriction was defined by a race-specific standard (Figure 5a). Growth restriction rates defined using the race-specific birth weight for gestational age standard showed a crossover with blacks having significantly higher growth restriction rates than whites below 39 weeks and significantly lower growth restriction rates at 39 weeks and over. For instance, rates of growth restriction as defined by the race-specific standard were significantly *lower *among blacks compared with whites at 40 weeks gestation (rate ratio 0.89, 95% confidence interval 0.88 to 0.91, p < 0.0001), despite a significantly *higher *perinatal mortality rate among blacks at 40 weeks gestation (rate ratio 1.43, 95% confidence interval 1.29 to 1.58, p < 0.0001). On the other hand, rates of gestational age-specific growth restriction were qualitatively congruent with patterns of gestational age-specific perinatal mortality when growth restriction among blacks and whites was defined using a single birth weight for gestational age standard (Figure 5b). For example, at 40 weeks gestation, the significantly *higher *rate of perinatal death among blacks was consistent with the significantly *higher *rate of growth restriction seen among blacks when a single standard was used to define growth restriction (rate ratio for growth restriction at 40 weeks among blacks vs whites 2.06, 95% confidence interval 2.04 to 2.09, p < 0.0001). Growth restriction (based on a single standard for both races) and perinatal mortality rates were substantially higher among births to black mothers as compared with births to white mothers at all gestational ages (Figure 5b).

## Discussion

We have confirmed previous observations that birth weight-specific perinatal mortality rates among male and female births exhibit a puzzling crossover paradox [1]. Gestational age-specific perinatal mortality rates among males and females were similar when mortality rates were calculated per convention (using total births at a particular gestation for calculating the perinatal mortality rate). On the other hand, use of the fetuses at risk formulation [15-19,41-44] showed that males have a consistently higher perinatal mortality rate at all gestational ages. Further, our study shows that gestational age-specific growth restriction and perinatal mortality rates both increase with advancing gestational age. Gestational age-specific rates of growth restriction among males and females are qualitatively congruent with gestational age-specific perinatal mortality patterns when growth restriction rates are based on a sex-specific birth weight for gestational age standard. Use of a single standard for males and females results in a gestational age-specific pattern of growth restriction that cannot be reconciled with gestational age-specific differences in perinatal mortality among males and females.

In contradistinction, contrasts between whites vs blacks show that use of a single birth weight for gestational age standard for both races is justified, while the use of a currently available race-based standard is not defensible. Gestational age-specific growth restriction patterns among whites vs blacks based on a single standard correspond qualitatively to patterns of gestational age-specific perinatal mortality among whites and blacks (Figure 5).

Birth weight for gestational age standards are modeled after infant and child growth standards and assume that fetal growth restriction occurs at a constant rate throughout pregnancy. This assumption is implicit in the use of the same, fixed cut-off (eg., the 3^rd ^percentile or the 10^th ^percentile cut-off of birth weight for gestational age) for identifying fetal growth restriction at all gestational ages. Our findings challenge the former assumption and show that in fact fetal growth restriction rates are better viewed as increasing with advancing gestational age (Figures 4 and 5). This contention is supported by the finding that gestational age-specific growth restriction rates follow the pattern of gestational age-specific perinatal mortality rates. Recent studies which show that the incidence of hypertensive disorders and chorioamnionitis increases with increasing gestational age provide at least a partial explanation for the gestational age-dependent rise in fetal growth restriction and perinatal mortality rates [45,46].

Table 3 shows that differences in stillbirth rates between males and females are smaller than differences in early neonatal mortality rates. The phenomenon of higher neonatal mortality differentials (relative to stillbirth differentials) between males and females has been previously noted [1] and is probably a consequence of obstetric intervention. Obstetric intervention (i.e., early delivery through labor induction and/or cesarean delivery) is typically prompted by signs of fetal compromise and will be more likely among pregnancies with male fetuses given the male fetuses' greater biological vulnerability. Such intervention leads to a reduction in the stillbirth differential, while having a smaller (or the opposite) effect on neonatal mortality differences between males and females. This explanation is supported by the higher rates of labor induction (and labour induction and/or cesarean delivery) observed among pregnancies with male fetuses (Figure 3b). Differences in rates of congenital anomalies that are lethal after birth and more frequent in males (eg., X-linked recessive conditions) may partly contribute to this phenomenon as well.

The slightly higher rate of gestational age-specific labor induction/cesarean delivery among males relative to females is encouraging since it suggests that the small mortality risk difference between males and females is already being addressed by modern obstetric practice (despite male sex not being formally identified as a factor in decision making related to obstetric intervention). This may be a consequence of the use of sex-specific birth weight for gestational age standards or sex-specific ultrasound-based fetal growth standards and, as mentioned, probably also reflects higher rates of suspected fetal compromise among pregnancies with male fetuses. Despite the marginally higher rates of labor induction among pregnancies with male fetuses, however, mortality differences persist. Research should be directed at ascertaining whether excess neonatal mortality among males can be successfully reduced through explicit recognition of male sex as a factor for altering the threshold for obstetric intervention.

Although contemporary birth weight for gestational age standards have substantial face validity [1,47,48], their development would benefit from greater empirical support and validation. For instance, it should be feasible to refine standards based on empirically observed (cause-specific) patterns of birth weight-specific perinatal mortality and serious neonatal morbidity (at each gestational age). This would represent an improvement over current standards which rely heavily on theoretical assumptions (eg., normality of birth weight at any given gestational age) and insufficiently on relevant empirical information (namely, perinatal morbidity and mortality related to growth restriction). Such cross-sectional information cannot address fetal growth in continuing pregnancies, however; the latter requires longitudinal information which is ideally obtained through ultrasonographic measurements. On the other hand, estimation of fetal weight through ultrasonography [31,49] needs to be improved [50,51] and diagnostic methods for identifying fetal growth restriction have tended to rely on other indicators of growth restriction besides estimated fetal weight.

Our study has limitations that are typical of studies that use large data bases. Errors in gestational age information are inevitable, although the magnitude of these errors is likely to be similar among male and female births. The overall rate of missing gestational age was low, however (0.9 percent among white live births and 0.8 percent among black live births). Our estimates of gestational age-specific fetal growth restriction rates are approximate. Ideally, estimation of the incidence of fetal growth restriction requires identification of fetal growth restriction on a longitudinal basis among continuing pregnancies [18]. The alternative measure of gestational age-specific growth restriction employed in our study represents an index of 'revealed' fetal growth restriction [18]. This approximation is unlikely to be a factor that seriously distorts patterns of gestational age-specific growth restriction since faltering of fetal growth typically leads to a spontaneous delivery or delivery following obstetric intervention. Other potential limitations of our study include the use of gestational age information on stillbirths. The gestational age at delivery of a stillbirth typically overestimates the gestational age at the time of fetal death, although this difference is unlikely to be large in recent years. Further, both male and female stillbirths would have been affected by this measurement error to a similar extent.

## Conclusion

The fetuses at risk approach resolves the paradox of intersecting perinatal mortality curves. Male births have higher rates of gestational age-specific perinatal mortality than female births. There is empirical justification for using sex-specific standards of birth weight for gestational age since gestational age-specific growth restriction patterns based on such standards correspond qualitatively with gestational age-specific perinatal mortality patterns. On the other hand, a single birth weight for gestational age standard for whites and blacks in the United States appears more appropriate than currently available race-specific standards since gestational age-specific growth restriction patterns among blacks and whites (based on a single standard) are qualitatively congruent with gestational age-specific patterns of perinatal mortality.

## Competing interests

The author(s) declare that they have no competing interests.

## Authors' contributions

KSJ proposed the study, carried out the analyses and drafted the manuscript. The results of the analyses were presented and discussed at a meeting of the Fetal and Infant Health Study Group of the Canadian Perinatal Surveillance System. All authors contributed to revising the manuscript for intellectual content. All authors read and approved the final version.

## Pre-publication history

The pre-publication history for this paper can be accessed here:


